# A Practical Method to Implement Strain-Level Metagenomics-Based Foodborne Outbreak Investigation and Source Tracking in Routine

**DOI:** 10.3390/microorganisms8081191

**Published:** 2020-08-05

**Authors:** Florence E. Buytaers, Assia Saltykova, Sarah Denayer, Bavo Verhaegen, Kevin Vanneste, Nancy H. C. Roosens, Denis Piérard, Kathleen Marchal, Sigrid C. J. De Keersmaecker

**Affiliations:** 1 Transversal activities in Applied Genomics, Sciensano, 1050 Brussels, Belgium; florence.buytaers@sciensano.be (F.E.B.); assia.saltykova@sciensano.be (A.S.); Kevin.Vanneste@sciensano.be (K.V.); nancy.roosens@sciensano.be (N.H.C.R.); 2 Department of Plant Biotechnology and Bioinformatics, Ghent University, 9000 Ghent, Belgium; Kathleen.Marchal@ugent.be; 3 National Reference Laboratory for Shiga Toxin-Producing *Escherichia coli* (NRL STEC), Foodborne Pathogens, Sciensano, 1050 Brussels, Belgium; sarah.denayer@sciensano.be (S.D.); bavo.verhaegen@sciensano.be (B.V.); 4 National Reference Center for Shiga Toxin-Producing *Escherichia coli* (NRC STEC), Department of Microbiology and Infection Control, Universitair Ziekenhuis Brussel (UZ Brussel), Vrije Universiteit Brussel (VUB), 1090 Brussels, Belgium; denis.pierard@uzbrussel.be; 5 Department of Information Technology, IDlab, IMEC, Ghent University, 9000 Ghent, Belgium; 6 Department of Genetics, University of Pretoria, 0001 Pretoria, South Africa

**Keywords:** metagenomics, SNP analysis, outbreak, food surveillance, whole genome, STEC

## Abstract

The management of a foodborne outbreak depends on the rapid and accurate identification of the responsible food source. Conventional methods based on isolation of the pathogen from the food matrix and target-specific real-time polymerase chain reactions (qPCRs) are used in routine. In recent years, the use of whole genome sequencing (WGS) of bacterial isolates has proven its value to collect relevant information for strain characterization as well as tracing the origin of the contamination by linking the food isolate with the patient’s isolate with high resolution. However, the isolation of a bacterial pathogen from food matrices is often time-consuming and not always successful. Therefore, we aimed to improve outbreak investigation by developing a method that can be implemented in reference laboratories to characterize the pathogen in the food vehicle without its prior isolation and link it back to human cases. We tested and validated a shotgun metagenomics approach by spiking food pathogens in specific food matrices using the Shiga toxin-producing *Escherichia coli* (STEC) as a case study. Different DNA extraction kits and enrichment procedures were investigated to obtain the most practical workflow. We demonstrated the feasibility of shotgun metagenomics to obtain the same information as in ISO/TS 13136:2012 and WGS of the isolate in parallel by inferring the genome of the contaminant and characterizing it in a shorter timeframe. This was achieved in food samples containing different *E. coli* strains, including a combination of different STEC strains. For the first time, we also managed to link individual strains from a food product to isolates from human cases, demonstrating the power of shotgun metagenomics for rapid outbreak investigation and source tracking.

## 1. Introduction

Food contaminations with pathogens are a major burden on our society, affecting an estimated 600 million people a year and impacting socioeconomic development at various levels [1]. Microbial contaminations include bacteria, viruses, or parasites and regularly result in extensive outbreaks as foodstuffs can be processed and traded at a large scale. In case of foodborne outbreak investigation, the microbiological analysis of the probable responsible food vehicle is performed at two levels and consists of the detection of the pathogen, followed by the association of the food vehicle to the human cases using typing of the food isolate. The fast and accurate source attribution allows to remove the product from the market and limit its impact on the population. For the detection of bacterial pathogens in food, the European Regulation (CE) 2073/2005 refers to ISO standards, although alternative methods are allowed if their performance has been demonstrated to be equivalent. Based on the symptoms of the human case, a set of pathogens is looked for through stepwise cultures on selective media and if relevant, the targeting of specific genes with real-time polymerase chain reactions (qPCRs) to characterize the strain. If the contaminant is successfully isolated, for some, it is characterized with Pulsed-Field Gel Electrophoresis or Multiple-Locus Variable Number Tandem Repeat Analysis for relatedness [2]. However, in the last decade, whole genome sequencing (WGS) of the isolate has been proposed as a higher resolution alternative for the full characterization of the micro-organisms [3,4]. This approach allows the detection of all genes present on the bacterial genome in just one test as well as phylogenetic analysis to link cases of food and human origin at the single nucleotide level. This resulted in recommendations from the European Centre for Disease Prevention and Control (ECDC) and the European Food Safety Authority (EFSA) to implement WGS on isolates in Europe for surveillance and outbreak investigation for a short list of priority pathogens and diseases [5,6,7].

However, the isolation of bacteria for the conventional method is a time-consuming process that is not always straightforward nor successful [8]. In that case, the outbreak investigation cannot be resolved at the microbiological level. Although qPCR-based detection methods of the food matrices, i.e., without isolation of the pathogen, can suggest the potential presence of the contaminant, it is not possible to link it back to the human cases. Sequencing methods with sufficiently high resolution that do not require isolation could solve this issue. A shotgun metagenomics approach consists in the direct sequencing of all DNA present in a sample. This gives an overview of the genomic composition of all cells in the sample, including the food source itself and the microbial community. This novel approach promises the detection of pathogens present in the sample without the need for isolation, avoiding problems linked to viable but non-culturable or difficult to isolate contaminants, and even circumventing the need for *a priori* knowledge about the causative agent [9,10]. DNA sequencing may also allow, if a sufficient depth can be obtained, to have the complete genetic information about the pathogen [10], to the single nucleotide polymorphism (SNP) level of accuracy. However, the challenge remains to correctly attribute each sequenced read to the appropriate strain with bioinformatics tools, in the presence of abundant host-originating reads, for a characterization of the pathogen’s genome, including the determination of which (virulence, serotyping…) genes are occurring on the same genome. The choice of the DNA extraction procedure might affect the quality of the obtained DNA as well as the proportion of the species including the host and the pathogen’s DNA. Some studies have previously researched the performances of several kits for metagenomics analysis on feces [11,12], but this has not yet been done for food. Another hurdle for the metagenomics analysis of food is the presence of the pathogens at very low abundances and the heterogeneity of the contamination in the food product. An enrichment of the target, already performed using the conventional microbiological methods, appears necessary. In previous metagenomics studies [13,14], different enrichment durations have been tested with several selective broths, and the possibility to use a random DNA amplification to replace the natural growth of the bacteria has been proposed. Researchers have demonstrated the potential of short reads shotgun metagenomics to identify bacteria in naturally contaminated or spiked food samples to a species- or even strain-level precision [15,16] or to characterize the pathogen by the detection of functional characteristics such as the presence of virulence genes [17,18]. A metagenomics method has also shown its potential in feces to detect multiple pathogens in one sample [19] and even multiple strains of the same species [20], but this has not yet been achieved at the lower level of contamination observed in food. The detection of multiple pathogens, even from the same species, would represent an added value to metagenomics compared to all traditional analyses requiring isolation, for which commonly only one isolate is further characterized. The currently available studies rather stayed in the research laboratory setting. However, a method used for routine practices in reference and routine laboratories requires following the guidelines of the current regulations or achieving results with at least similar performances, with a standard protocol for sample preparation that can be applied to a range of different food matrices. Therefore, a thorough validation is necessary upon its implementation.

The application of a metagenomics workflow to the issue of foodborne outbreaks could be particularly useful to circumvent the need for isolation in conventional methods and get a faster response in case of outbreak investigation. The Shiga toxin-producing *Escherichia coli* (STEC) is a particularly challenging pathogen to analyze with such an approach as its minimal infectious dose is very low, which is defined as 10 colony-forming units (CFU) [21], and non-pathogenic *E. coli* are vastly represented in the environment and in food often associated with STEC contamination [22]. Therefore, it is difficult to differentiate various strains in a sample and infer the virulence genes to its corresponding genome, to characterize the specific *E. coli* pathotype, and hence its potential danger for human, based on the presence of specific virulence genes. STEC is a zoonotic disease that is mainly contracted through food consumption, but it is also related to animal contact, human-to-human contact, and water or soil absorption [1]. It is a Gram-negative bacteria that is defined as a pathogen by its capability to produce one of two types of Shiga toxins, which are coded in a prophage containing the *stx1* or *stx2* genes [23]. Indeed, STEC bacteria share about 75% of their genome with non-pathogenic *E. coli* [24], and they acquire their toxicity through phages, which can also be present in food [25]. Another virulence factor of interest is the *eae* gene, which is present on the chromosome, and related to the production of intimin, a protein causing cell attachment to the intestinal wall. Using the presence of one or more of these genes, STEC are currently detected in routine laboratories through ISO/TS 13136:2012 [23]. STEC can cause bloody diarrhea that can lead to a hemolytic uremic syndrome (HUS) and even death. The severity of the disease can be predicted based on the subtype of the *stx1* and *stx2* genes, as well as the detection of other virulence factors such as the *eae*, *aaiC,* or *aggR* genes causing aggregative adherence to the intestinal mucosa of the host, or the *ehxA* gene responsible for the production of hemolysin [26,27]. However, this is not taken into account in the current regulations or international methods and requires extra qPCR tests or the sequencing of (parts of) the isolate’s genome. ECDC and EFSA have recommended the use of WGS to characterize this pathogen [5,7], but the acquisition of isolates is not always straightforward. Some recent STEC outbreaks have stressed the arduousness for an accurate source attribution due to difficulty in isolating [28]. Therefore, it could strongly benefit from a metagenomics approach.

We present here a metagenomics workflow for the full characterization of STEC in food matrices using short reads sequencing. Our workflow was developed by testing different laboratory methods on minced beef meat spiked at the lowest infectious dose. Different enrichment and DNA extraction methods were tested in order to define a practical workflow that can be implemented in a routine setting. We evaluated the performances of the different sample preparations for the full outbreak-like characterization of STEC spiked in this complex food matrix by comparing the results obtained with a metagenomics analysis to the results obtained following the current official conventional methodology. Our bioinformatics workflow was set up in order to obtain the same output as expected from the conventional methods, i.e., the detection of *Escherichia* spiked in the sample, which is predicted here through taxonomic classification, and the prediction of the presence and severity of a pathogenic *E. coli* based on the detection of virulence factors in the sequenced reads. The genome of the STEC was then inferred, corresponding to obtaining an isolate’s genome. It was characterized through gene detection and SNP phylogeny, in order to evaluate relatedness to other cases from human and food origin, as would be expected from routine analysis. Our analysis also went one step further by testing the selected workflow on samples of fresh goat cheese simultaneously spiked with two different STEC serotypes but possessing identical virulence genes.

## 2. Materials and Methods

### 2.1. Spiked Sample Preparation

The experiments on beef were conducted with one strain of STEC selected from the collection of the Belgian National Reference Laboratory (NRL) (TIAC 1152, O157:H7, *stx1+, stx2+, eae+*). This strain was related to an outbreak in Limburg in 2012 [27], and its genome was previously sequenced in another study [29]. The inoculum preparation and artificial contamination of the food matrix was carried out according to Barbau-Piednoir et al. [30]. Briefly, a STEC culture in Brain Heart Infusion (BHI) broth was diluted to obtain an OD_600nm_ of 1, which was then diluted to 10^−7^ in buffered peptone water. An enumeration of 100 µl of the dilution was performed in triplicate on nutrient agar plates incubated for 18 ± 2 h at 37 °C (see count in Supplementary Materials Table S1). Organic minced beef meat was purchased at a local store (composition: 99.7% organic beef, natural aroma; nutritional values per 100 g on the package: energy: 464 kJ, total fat: 2 g, carbohydrates: 0 g, proteins: 22 g, salt: 1.1 g). A test portion of 25 g was 1/10 diluted in buffered peptone water (BPW), homogenized, and subsequently contaminated with 10 µl of the dilution 10^−7^, corresponding to the minimal infective dose of STEC (5–10 CFU). This artificial contamination was repeated three times (biological triplicates, representing biologically distinct samples accounting for random biological variation). One sample was not artificially contaminated (the “Blank”, Bk).

The same procedure was followed on fresh organic goat cheese from raw milk purchased at a local store (composition and nutritional values were not specified on the package. Average values for goat cheese macronutrients per 100 g are proteins: 21.58 g, carbohydrates: 0.12 g, total sugars: 0.12 g, total fibers: 0 g, total fat: 29.84 g, [31]) with the strains TIAC 1220 (O145:H28, *stx1*+, *eae*+) and TIAC 1878 (O103:H2, *stx1*+, *eae*+) from the Belgian NRL. The strains were spiked separately and co-spiked at a level of 5–10 CFU in a 25 g food matrix. For milk and dairy products, the diluent as recommended in ISO/TS 13136:2012 was used [23]: modified tryptic soy broth with the addition of acriflavin (12 mg/L) for inhibition of the growth of Gram-positive bacteria.

**Figure 1:**
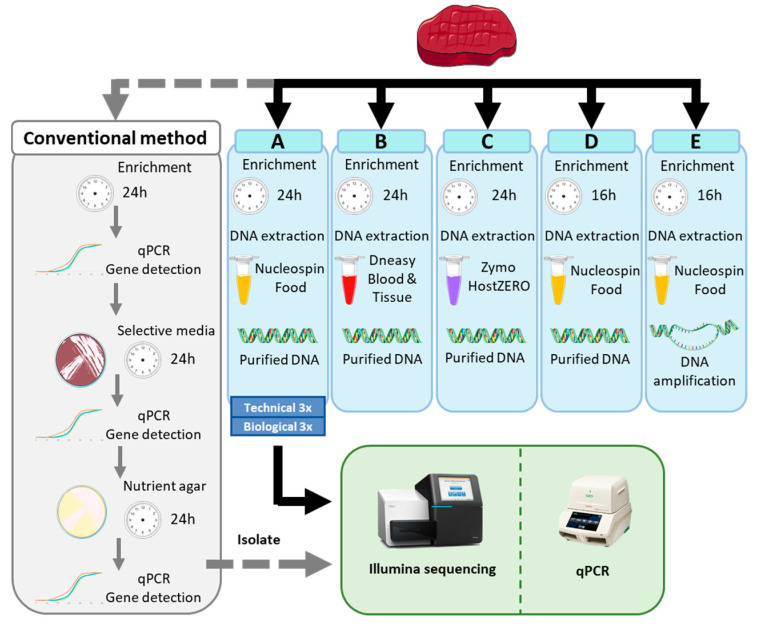
Presentation of 5 different workflows for the preparation of metagenomics samples of spiked beef (light blue) and the conventional method for Shiga toxin-producing *Escherichia coli* (STEC) detection and characterization based on several steps of qPCR and isolation on selective media (ISO/TS 13136:2012, grey). The extracted DNA (amplified or not, from metagenomics samples or isolate) is tested for quality control (DNA purity, integrity, concentration) before sequencing on the Illumina MiSeq in parallel to a qPCR check for the presence of *stx* genes (green).

The samples were incubated for 24 h at 37 °C without shaking. The third biological replicate of the spiking was incubated for 16 h in the same conditions (methods D and E, Figure 1). After enrichment, 1 mL of the culture was centrifuged at 6000× *g* for 10 min, and the cell pellets were stored at −20 °C until DNA extraction. No fat layer was observed at the surface of the centrifuged beef, but a fat layer was observed after centrifugation of the goat cheese and was manually removed before DNA extraction, following Volk et al. [32].

### 2.2. DNA Extract Preparation 

Three commercial kits were used for the DNA extraction from beef samples. This was done on one of the biological replicates for the three kits: the Nucleospin Food (Macherey-Nagel, Düren, Germany, methods A, D, E, Figure 1), the DNeasy Blood and Tissue (Qiagen, Hilden, Germany, method B, Figure 1), and the HostZERO Microbial DNA kit (Zymo Research, Irvine, CA, USA, method C, Figure 1). DNA from all blank beef samples was extracted with the Nucleospin Food DNA extraction kit. The protocol was followed according to the manufacturer’s instructions on cell pellets. The elution buffer of the DNeasy Blood and Tissue was replaced by TrisHCl 10mM. The Nucleospin Food extraction was repeated on all biological replicates. Technical triplicates were produced for the DNA extraction of the third biological replicate to account for the variability due to the extraction protocol. One DNA extract of the beef sample enriched for 16 h and extracted with the Nucleospin Food kit was amplified using phi 29 DNA polymerase (ThermoFisher scientific, Waltham, MA, USA) according to the manufacturer’s instructions (method E, Figure 1).

Extraction blanks (extraction of water instead of the sample) were prepared for the three kits. Although no DNA could be detected using a Nanodrop 2000 (Thermo Fisher Scientific, Waltham, MA, USA), Qubit 3.0 Fluorometer (Thermo Fisher Scientific, Waltham, MA, USA), and 4200 TapeStation (Aglient, Santa Clara, CA, USA), they were included in metagenomics runs. The extraction blanks had very few reads, corresponding to less than 1% of the amount of reads sequenced per spiked beef samples (data not shown). The *Escherichia* genus was not detected in any of the reads from the blanks of the different kits after analysis using Kraken2 [33]. Therefore, it was concluded that none of the extraction kits contained DNA that could impact the results of our analysis.

The goat cheese samples were handled according to workflow A (incubation of 24 h and DNA extraction with Nucleospin Food) as described above.

The quality and quantity of all DNA extracts were evaluated using the Nanodrop 2000 (Thermo Fisher Scientific, Waltham, MA, USA), Qubit 3.0 Fluorometer (Thermo Fisher Scientific, Waltham, MA, USA), and 4200 TapeStation (Aglient, Santa Clara, CA, USA).

### 2.3. Real-Time Polymerase Chain Reaction Verification

The presence of STEC with the expected virulence pattern in blank and spiked samples was verified in all DNA extracts from food matrices using qPCR for the genes *uidA, eae, stx1,* and *stx2* as described by Barbau-Piednoir et al. [30].

### 2.4. Validation with ISO Method

The detection of STEC in the blank and spiked samples of all biological replicates of the experiment was validated in parallel following ISO/TS 13136:2012: qPCR on the crude extract, isolation on selective media (STEC colorex, CHROMagar), confirmation of the typical colonies by qPCR, isolation on nutrient agar, and confirmation with qPCR [23] (Figure 1). A detailed overview of the conventional methods used for the detection and characterization of STEC in food in the Belgian NRL can be found in Nouws et al. [34]. Then, re-isolated STEC colonies from nutrient agar plates were cultured overnight in BHI, and the DNA was extracted with the DNeasy Blood and Tissue kit according to the manufacturer’s protocol. The elution buffer was replaced by TrisHCl 10 mM.

### 2.5. Next-Generation Sequencing

All DNA extracts, including isolates, were further processed with the Nextera XT library preparation kit (Illumina, San Diego, CA, USA) before sequencing on the Illumina Miseq, generating paired-end 250-bp reads with the reagent kit v3, according to the manufacturer’s instructions. The samples were sequenced in three different sequencing runs, each containing libraries of 12 samples (Supplementary Materials Table S1).

### 2.6. Data Analysis

**Figure 2:**
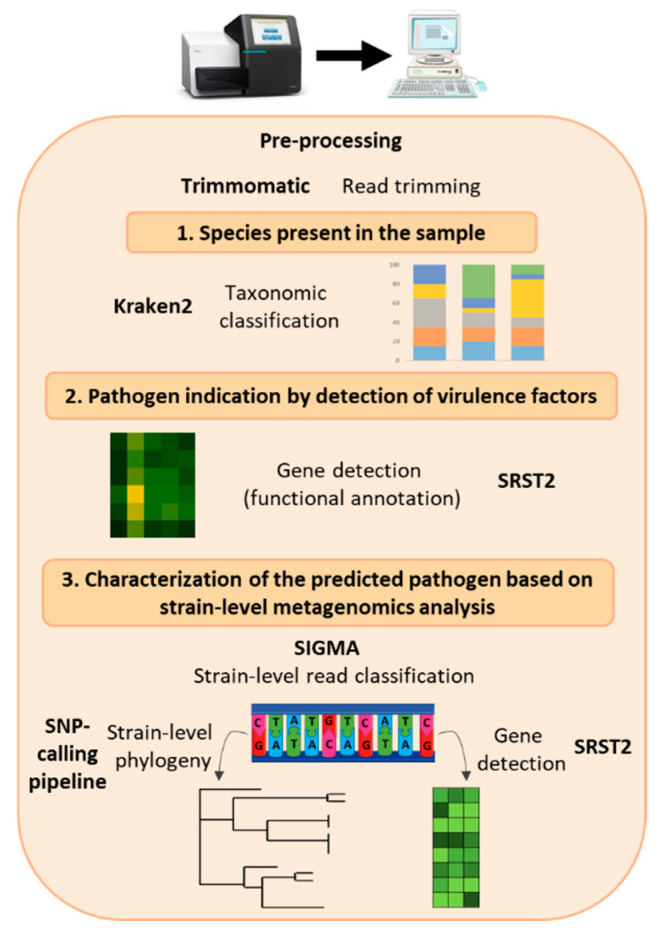
Presentation of the bioinformatics analysis for the characterization of STEC in samples using a metagenomics approach. After sequencing and pre-processing of the reads, first, the species in the sample are detected by a taxonomic classification tool (Kraken2); then, the presence of a pathogen in the sample is predicted based on the detection of virulence genes in the reads (SRST2), after which individual bacterial strains are inferred (Sigma) and characterized with gene detection (SRST2) and single nucleotide polymorphism (SNP) phylogeny (SNP-calling pipeline).

The sequence reads obtained for the isolates were further processed with the pipeline as described in Nouws et al. [29]. The number of reads sequenced per metagenomics sample is presented in Supplementary Materials Table S2. Then, raw reads were analyzed through a bioinformatics workflow presented in Figure 2. The reads were trimmed using Trimmomatic version 0.38.0 operating sliding window trimming averaged on 4 bases requiring an average quality of 20 [35] (Supplementary Materials Table S2). A taxonomic classification of the reads was conducted using Kraken2 version 2.0.7 [33] first with an in-house database of mammalian sequences containing the following genomes in order to filter out the host DNA: *Bos taurus* (GCF_000003055), *Capra hircus* (GCF_001704415), *Chlorocebus sabaeus* (GCF_000409795), *Mesocricetus auratus* (GCF_000349665), *Cavia porcellus* (GCF_000151735), *Equus caballus* (GCF_000002305), *Mus musculus* (GCF_000001635), *Rattus norvegicus* (GCF_000001895), *Ovis aries* (GCF_000298735), and *Sus scrofa* (GCF_000003025). Genomes were retrieved on 18/02/2019. Second, this was then followed by a classification on an in-house database of archaea, bacteria, fungi, human, protozoa, and viruses. This customized Kraken database was built using all available RefSeq “complete Genome” sequences of the targeted taxonomic groups downloaded from the RefSeq Genome (ftp://ftp.ncbi.nlm.nih.gov/genomes/refseq/) on 18/02/2019 [36]. This classification step was done using a two-step approach because a joint search against both databases requires computational resources beyond what is available for the Belgian NRL. Graphs were created on the classification results using ggplot2 in R. A gene detection was performed on all trimmed reads with SRST2 version 0.2.0 [37] on the databases of VirulenceFinder *E. coli* and shiga-toxin genes [38] and SerotypeFinder O type and H type [39] as accessed in January 2020, filtering genes covered at 80% and above with a maximum divergence of 20% (results presented in Supplementary Materials Table S2). Graphs of the depth of detection normalized to 1 million trimmed reads per sample were drawn using R and the library ComplexHeatmap [40]. Strain-level metagenomics analysis was performed using Sigma [41] following the method described by Saltykova et al. [42] with a database of 728 complete genomes of *Escherichia coli* from the National Center for Biotechnology Information (NCBI). Gene detection with SRST2 was performed on the classified reads corresponding to the individual *E. coli* strains with the same databases using a minimal coverage of 30% and maximal divergence of 20% as parameters. All genes detected in the strains with these parameters were considered present if they were detected with 80% coverage and identity in all reads from the sample, taking into consideration that part of the sequence might be lost in the read classification by Sigma. For phylogenetic analysis, SNP calling was carried out on the classified reads as previously described by Saltykova et al. [42], with *E. coli* O157:H7 str. Sakai (BA000007.2) as a reference. A matrix of SNP differences per million genomic positions covered was calculated and presented in Supplementary Materials Table S3. Maximum likelihood substitution model selection and phylogenetic tree inference were done with MEGA [43], using the NNI (nearest-neighbor-interchange) heuristic method, keeping all informative sites and using the bootstrap method with 100 replicates as a phylogeny test. The parameters of the model selected for the construction of each tree are presented in Supplementary Materials Table S4. iTOL [44] was used for the representation of the tree with percentage of reference genome covered and gene detection displayed as annotations on the side of the branches. The percentage of the reference genome represents the fraction of the genome that was suitable for the phylogenetic analysis and not the percentage of positions in the genome that were covered by reads (due to (imperfect) repeats excluded during SNP calling because of lower mapping quality). The strains used in the tree were sequenced for another study based on isolates from the National Reference Laboratory of STEC [29] (accession numbers: SRR10201483, SRR10201465, SRR10201452, SRR10201427, SRR10201416, SRR10201408, SRR10201398, SRR11816083, SRR11816082, SRR11816006, SRR11816065, SRR11816010, SRR11816005, SRR11816071, SRR11816075, SRR11816012, SRR11816073). The data from all metagenomics samples presented in this study can be accessed under BioProject PRJNA645436.

## 3. Results

### 3.1. Testing of 5 Sample Preparation Workflows for Metagenomics Analysis Applied on Spiked Beef

Different parameters were tested in parallel for the handling of spiked beef samples as presented in Figure 1, to investigate which workflow would be the most appropriate and performant for a strain-level metagenomics-based outbreak investigation in reference and routine laboratories. We evaluated the performances of 5 metagenomics sample preparation workflows, which differed by the DNA extraction kit used and enrichment method (16 versus 24 h enrichment, with or without a DNA amplification step) (Materials and Methods; Figure 1). The outcome of metagenomics analysis of the samples was compared to the conventional methods involving isolation of the pathogen used in the National Reference laboratories, ISO/TS 13136:2012, followed by WGS of the isolate. Our metagenomics data analysis (Figure 2) aimed at obtaining at least the same results as the conventional methods, i.e., the detection of a STEC, serotype, and three virulence genes (*eae*, *stx1*, *stx2*), and the determination of relatedness to outbreak cases without isolation.

#### 3.1.1. Comparison of the Experiment with Conventional Methods

The blank and spiked samples were tested following the conventional methods (i.e., ISO/TS 13136:2012), which are currently used in the National Reference laboratories (Supplementary Materials Table S5). All samples gave the expected results according to the spiking: detection of the *stx* and *eae* genes with qPCR in the crude extract (Supplementary Materials Table S5) and in isolates after consecutive culture steps (use of selective media and isolation of typical colonies on nutrient agar, Figure 1) [34], except in the blanks. Furthermore, sequencing of the isolates obtained from these samples with the conventional methods, an analysis recommended by EFSA but not included in the ISO, allowed the detection of the expected virulence profile and serotype in the sequencing reads. The isolate could be related to the other outbreak cases including patient’s strains in a phylogenetic analysis (see phylogenetic analysis below, Section 3.1.3, Section 3.1.4 and Section 3.1.6). These results confirmed the correct course of the spiking experiment.

#### 3.1.2. Analysis of Blank Beef Samples

The absence of STEC in the food matrix was investigated using qPCR (Supplementary Materials Table S1) as well as shotgun metagenomics analysis (Figure 2) of the DNA extracts of the blanks. The *uidA* gene, an indicator for the presence of *E. coli*, was detected by qPCR in the DNA extracts of all blank samples, including the non-enriched blank where it was observed at a high quantification cycle (Cq 33). Some variation was present between the biological replicates of the enriched blank: the first biological replicate showed lower levels of *E. coli* (Cq 22) compared to the two others (Cq < 20). 

**Figure 3:**
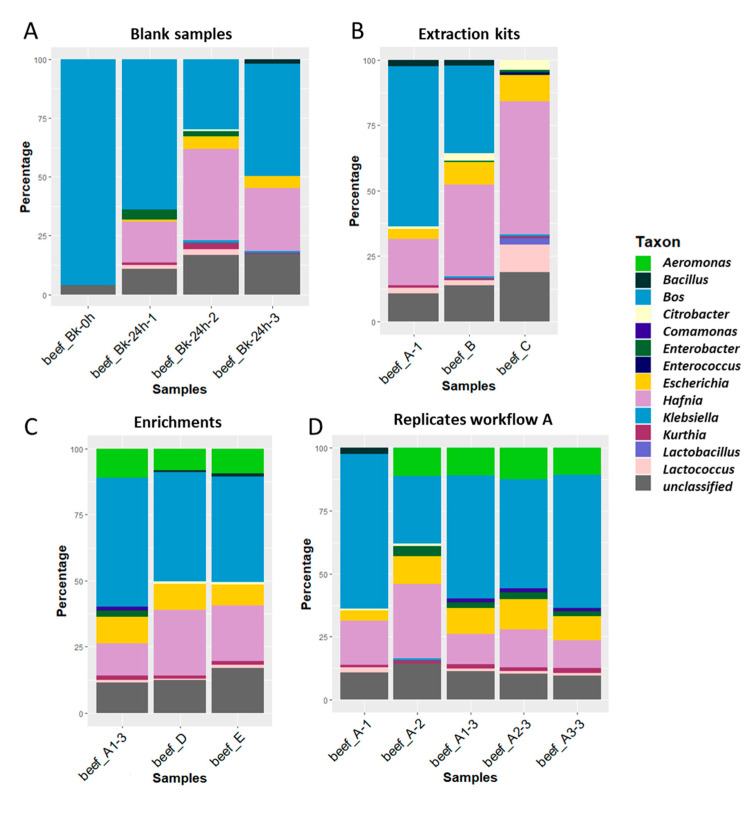
Percentages of reads classified to the genus level using Kraken2 (taxonomic classification tool) from beef samples with in-house databases of mammals, archaea, bacteria, fungi, human, protozoa, and viruses. Light blue represents the proportion of “*Bos*” corresponding to beef reads. Yellow represents the presence of “*Escherichia*” in the sample. The reads that could not be classified to the genus level for mammals, archaea, bacteria, fungi, human, protozoa, or viruses are represented in gray. (**A**) Blank meat samples; Bk-0h—non-enriched blank; BK-24h—non-spiked meat sample enriched for 24 h, 1–3 biological replicates. (**B**) Extraction kits; workflow A—Nucleospin Food, workflow B—DNeasy Blood & Tissue and workflow C—Zymo HostZERO. (**C**) Enrichment times; workflow A—24 h culture enrichment, workflow D—16 h culture enrichment, workflow E—16 h culture enrichment, extraction followed by DNA amplification using phi 29 DNA polymerase; all extracted with Nucleospin Food kit. (**D**) Biological and technical replicates of workflow A. Small differences in the detected species shown in panels A, C, and D can be explained by the heterogeneity of the samples and biological variation, as different replicates of the experiment were used.

Figure 3A presents the taxonomic distribution to genus level in each blank sample. The non-enriched blank of the first biological experiment (beef_Bk-0h) presented 96% of reads classified as *Bos*, corresponding to the host food matrix (beef meat). No bacterial species could be detected without enrichment. After enrichment, the three biological replicates of the blank (beef_Bk-24h-1, -2, and -3) harbored on average about 50% of *Bos* reads (47%, 29%, and 64% respectively), while the other reads were distributed between bacteria naturally present in the beef meat. Of these, *Escherichia* was detected in each biological replicate as one of the major taxons, indicating that the bacteria of this genus were naturally present in the beef matrix. Therefore, the sequencing results confirmed the qPCR results for the *uidA* gene obtained for the food mix crude extract analyzed with the conventional method (Supplementary Materials Table S5), and the qPCR results on the DNA extracts of this food mix (Supplementary Materials Table S1). *Escherichia* was detected with less reads in the first biological replicate (1%), confirming the qPCR observations (higher Cq, Supplementary Materials Table S1). *Hafnia* and *Kurthia* were also detected in all blanks. These species are commensal in ruminants and often detected in meat products [45,46]. Although *Hafnia alvei* has been described in rare cases as an opportunistic pathogen to humans, it is not considered to contribute to meat spoilage or pose a risk to human health [47,48]. We did not observe an issue with the integrity of the meat when the analysis was conducted (within the expiry date). Some other less represented genera varied between the samples (i.e., *Klebsiella, Enterobacter, Citrobacter, Bacillus,* and *Lactococcus*).

**Figure 4:**
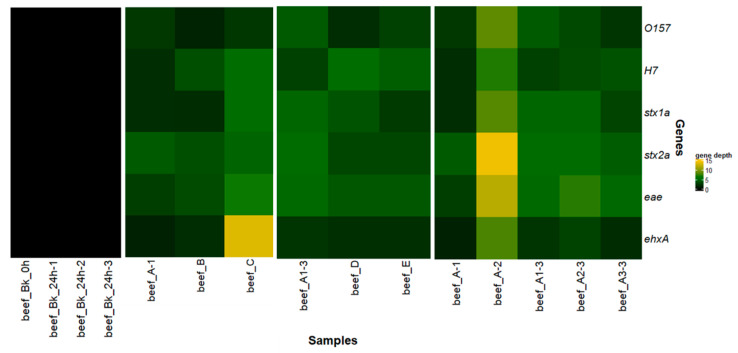
Gene depth per million trimmed reads per sample for the detection of genes encoding for serotype O157:H7 (*wzx* and *fliC* genes) and the *stx1a*, *stx2a*, *eae,* and *ehxA* virulence genes (5 genes from ISO/TS 13136:2012 and *ehxA* present on plasmid pO157) with more than 80% query coverage and 80% identity in all reads for beef samples processed with different workflows A-B-C-D-E, and in biological (A-1, A-2, A-3) and technical replicates (A1-3, A2-3, A3-3) of workflow A. Increasing depth (per million trimmed reads) is represented in shades of green to yellow according to the color gradient in the legend.

The genes *stx1, stx2,* and *eae* were not detected in any of the blank samples with qPCR (Supplementary Materials Table S1) nor with gene detection in the sequenced reads (Figure 4, Supplementary Materials Table S2). These observations, as well as the taxonomic classification, indicate that endogenous non-pathogenic *E. coli* were already present in the beef sample prior to spiking, as detected by qPCR. Accordingly, no STEC strains could be inferred for relatedness according to our bioinformatics protocol (Figure 2), although reads from non-pathogenic endogenous *E. coli* could be obtained.

#### 3.1.3. Testing of 3 DNA Extraction Kits for the Spiked Beef Samples

The DNA extraction of spiked beef enriched for 24 h was conducted with 3 different commercial kits (methods A, B, C) on the same sample (first biological replicate). The three DNA extracts tested positive for the presence of *E. coli* DNA, as indicated by the *uidA* qPCR assay, with a Cq of 20 for workflow A, 19 for workflow B, and 18 for workflow C (Supplementary Materials Table S1).

After taxonomic classification, *Escherichia* reads could be retrieved in all DNA extracts from the spiked and blank samples (Figure 3B), and in higher proportions than in the corresponding blank (beef_Bk_24h-1, Figure 3A), as expected from the artificial addition of the STEC strain. No reads were classified as *Bos* in the sample extracted with HostZero (workflow C), demonstrating the efficiency of the kit to remove the DNA of eukaryotic cells. Meanwhile, 9.8% of the reads of the HostZero DNA extract were classified as *Escherichia*, while a very high percentage of all bacteria were classified as *Hafnia*. The use of the two other kits (workflows A and B) led to similar profiles with some variations in the percentages of the taxons. *Escherichia* was correctly detected in those DNA extracts (4% for workflow A and 8.5% for workflow B).

The serotype and the virulence (based on the detection of 6 genes) were correctly determined after extraction with the three different extraction kits (Figure 4 and Supplementary Materials Table S2), confirming the results of the qPCR (Supplementary Materials Table S1). Even the subtyping of the *stx1* and *stx2* virulence factors could be achieved. After normalization per million trimmed reads, *ehxA*, a gene present on the pO157 plasmid, was mapped with less reads (lower depth) than the ones present on the chromosome for workflows A and B, but with more reads for workflow C. The DNeasy Blood and Tissue kit (workflow B) had overall less reads mapping to all studied genes.

**Figure 5:**
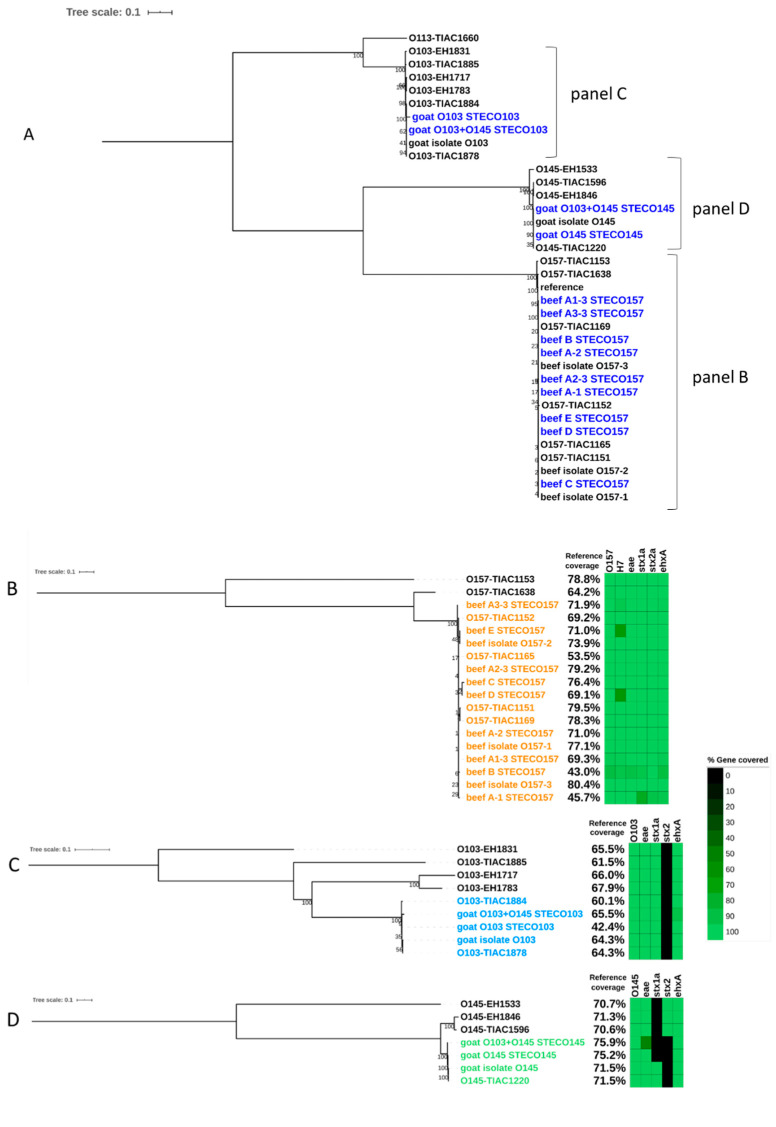
**(A**) SNP-based phylogenetic tree of STEC strains inferred from metagenomics samples (dark blue) and of sequenced isolates (black). Reference: *E. coli* O157:H7 str. Sakai (BA000007.2). Beef/goat isolate: STEC isolate obtained after following the conventional method on the prepared spiked samples. (**B**) Phylogenetic tree of the STEC O157 with percentage of the reference genome covered and gene detection in the strains. Orange: closely related strains from the outbreak cluster. (**C**) Phylogenetic tree of the STEC O103 with percentage of the reference genome covered and gene detection in the strains. Blue: closely related strains. (**D**) Phylogenetic tree of the STEC O145 with percentage of the reference genome covered and gene detection in the strains. Green: closely related strains. The scale bar represents nucleotide substitution per 100 nucleotide site. Node values represent bootstrap support values.

Strain-level metagenomics analysis performed on the WGS data of complex food matrices indicated that the spiked samples contained more than one *E. coli* strain. For all samples, one strain was identified as STEC by the detection of *stx* genes and was used in the phylogenetic analysis. The other strains are considered as endogenous *E. coli*. For all three DNA extraction kits, the detected STEC strain clustered with food (TIAC 1151, 1152) and human (TIAC 1169, 1165) isolates of the Limburg outbreak with 0 to 1 SNPs difference per million genomic position (Supplementary Materials Table S3) and separated from non-outbreak isolates (TIAC 1153 and TIAC 1638) (Figure 5A,B) on the phylogenetic tree. All DNA extraction kits allowed the determination of the 6 genes (typing genes *wzx* and *fliC* corresponding to serotype O157:H7 and virulence genes *stx1*, *stx2*, *eae* and *ehxA*) in the reads of the STEC strain, confirming that it was the spiked strain. The subtype of the *stx* genes could also be obtained in the inferred strains. The percentage of the reference genome covered after DNA extraction of the same biological sample, presented on the side of the tree in Figure 5B, was higher with workflow C (HostZero extraction) and lower for workflow B (DNeasy Blood & Tissue), but all were in line with the percentages observed for isolates, indicating that a sufficient sequencing depth could be achieved for the spiked strain for a robust phylogenetic placement. The remaining *E. coli* strains detected in the metagenomics samples were also screened for the presence of virulence genes but resulted negative for the detection of *stx1*, *stx2*, eae, and *ehxA*, indicating that they represented the endogenous *E. coli* present on the food matrix. Therefore, these were not investigated further.

#### 3.1.4. Testing of Different Enrichment Procedures

To see whether the enrichment of 24 h could be shortened, workflow A (24 h) was compared on the same sample (third biological replicate) to an enrichment of 16 h (workflow D) and an enrichment of 16 h with the same DNA extraction kit (Nucleospin Food), followed by a DNA amplification using phi 29 polymerase (workflow E).

All extracts were positive for *uidA* tested in qPCR (Supplementary Materials Table S1). All three workflows showed comparable Cq values (A and D: 20, E: 23). Sequencing and taxonomic classification confirmed the low variation between the samples (Figure 3C). *Escherichia* was detected in the three DNA preparations and represented between 8% and 10% of the reads. The amplification of the DNA (workflow E) did not qualitatively affect the distribution of the species in the sample, but resulted in a higher amount of unclassified reads. Two low abundance species (*Comamonas* and *Enterobacter*) detected in the 24 h enrichment were not detected in the 16 h enrichment samples (D and E). This can be due to growth differences during the culture and/or incubation time, which were conducted in two separate bags.

qPCR on the virulence genes of interest (Supplementary Materials Table S1) gave similar results for the sample enriched for 16 h (workflow D) and the sample enriched for 24 h (workflow A). The sample processed with workflow E (16 h of enrichment and DNA amplification) had slightly higher Cqs for all genes tested. After sequencing, the 5 genes from the ISO standard and *ehxA* were detected in the DNA extracted from the food samples processed with the three different workflows (Figure 4). The time of enrichment did not impact the gene detection, as the depth (normalized per million trimmed reads) was in the same range for the food samples enriched for 16 or 24 h (Figure 4). The DNA amplification (workflow E) had similar results to the sample preparations without amplification (Figure 4 and Supplementary Materials Table S2).

After strain-level metagenomics analysis, one STEC strain was inferred from each metagenomics sample while other endogenous *E. coli* were present in the same samples. The obtained STEC strain could be related to the isolates from food and human origin of the same outbreak for the three workflows (Figure 5A,B) in a phylogenetic analysis. The obtained strain of each of the three workflows had 0 SNP difference per million genomic position with the outbreak isolates (Supplementary Materials Table S3) and shared similar SNP differences as these isolates have with the non-outbreak cases. All 5 genes from the ISO standard could be detected in the inferred genomes, as well as *ehxA*, although a lower coverage for the gene coding for type H7 was observed in the DNA extracted from the food samples enriched for 16 h (workflows D and E).

#### 3.1.5. Evaluation of the Performances of the Tested Metagenomics Workflows

As elaborated above, all workflows allowed a characterization of the pathogen after enrichment, comparable to the conventional method, but without prior isolation: i.e., detection of STEC in the sample, determination of the serotype and virulence factors of interest, and retrieval of the reads corresponding to the STEC strain to perform phylogenetic tracing back to the Limburg outbreak. However, some small drawbacks were noted. Workflow B, although easy to implement for an average cost, resulted in low depths for the detection of the genes of interest and a lower coverage of the genome for the reference strain. Workflow C yielded very good results for gene detection and linkage to the outbreak isolates as well, but it is more expensive compared to the other methods. Workflow E did not show sufficient added value of the DNA amplification to pursue this additional step. Workflows A and D consisted of DNA extraction with the Nucleospin Food kit, differing in the enrichment time (24 or 16 h). Overall, workflow A showed a good performance for gene detection and strain-level metagenomics analysis with a short hands-on time and low price per sample. Additionally, 24 h of enrichment followed by a fast DNA extraction protocol seemed to be the most practical during outbreak investigation and to be used in a reference or routine laboratory setting, as it is in line with the current ISO regulation (ISO/TS 13136:2012). Therefore, workflow A was selected for further analyses.

#### 3.1.6. Reproducibility of Workflow A

The reproducibility of the selected workflow A was verified using biological and technical replicates, representing the random biological variation due to the spiking and enrichment of the samples, and the variability due to the extraction protocol, respectively. After DNA extraction, the *uidA* gene was detected with similar Cq (19.34 to 20.46), as tested with qPCR (Supplementary Materials Table S1). After sequencing, the obtained reads were classified per taxon (Figure 3D). *Bos* accounted for approximately half of the reads in all samples, while *Kurthia, Hafnia,* and *Escherichia* were also present in every replicate. *Escherichia* was detected at various levels in all the spiked samples, roughly twice as much as in the corresponding blanks (Figure 3A). The increase of bacteria of this genus results from the addition of the STEC inoculum, introducing a new strain of this genus in the mix. The same species were detected in the three technical replicates (A1-3, A2-3, A3-3), and the difference in the species distributions was low. The biological replicates had more variation, and the first one presented the least reads classified as *Escherichia* in the blank and the spiked sample.

The genes of interest were detected in all replicates of the experiment using qPCR (Supplementary Materials Table S1) and in the sequenced reads (Figure 4). The gene *ehxA* was detected with a lower depth in all replicates. After normalization to a million trimmed reads, the first biological replicate of the experiment had overall lower depths for the 6 genes, while the second biological replicate showed higher amount of reads mapping to each gene of interest, but all genes could be detected, including the subtype of *stx1* and *stx2*, for all samples. This can be linked to the lower percentage of reads classified as *Escherichia* in the DNA extracted from this food sample (Figure 3A) as well as a higher Cq in qPCR (Supplementary Materials Table S1). The variation between technical triplicates was lower than that between the biological replicates.

Strain-level metagenomics analysis allowed the detection of a STEC strain in all replicates of the experiment. These strains clustered correctly with the isolates linked to the outbreak on the phylogenetic tree (Figure 5A,B) with 0 to 1 SNPs distance per million genomic positions (Supplementary Materials Table S3). In every case, the strain harbored the serotyping and virulence genes as used in ISO characterization, as well as *ehxA*, and covered about 70% of the reference genome, except for the first biological replicate that covered 45.7% of the reference genome.

### 3.2. Detection and Characterization of Two STEC Strains in Goat Cheese

The same metagenomics analysis was conducted on spiked goat cheese samples in parallel with the conventional method, as a reference. This matrix is known to be difficult to analyze due to its high fat content and complex bacterial community [32]. Additionally, we increased the complexity further by spiking two other STEC serotypes (O103 and O145) harboring identical *stx* and *eae* genes separately and in a co-contamination scenario (the two strains were spiked simultaneously). Sample preparation was conducted following the selected workflow A (enrichment of 24 h and extraction using Nucleospin Food, Figure 1), and the same bioinformatics analysis (Figure 2) was applied.

#### 3.2.1. Comparison of the Experiment with Conventional Method

The blank and spiked goat cheese samples were tested following the conventional methods (ISO/TS 13136:2012) to verify the spiking step. The blank goat cheese (not spiked) was negative for *uidA, eae, stx1,* and *stx2* with qPCR, and no isolate could be obtained (Supplementary Materials Table S5). All spiked samples could be completely characterized with the conventional method, including the co-spiked sample: i.e., the virulence genes were detected in the enriched food matrix (*eae, stx1*) with qPCR, the isolation of the STEC strain(s) on selective media, their isolation on nutrient agar, and the confirmation of obtaining of an STEC with qPCR was achieved. After sequencing of the obtained isolates with WGS, the expected virulence profile and serotype were obtained for both strains based on the corresponding gene detection in the sequenced reads. Then, the isolates were placed in phylogenetic trees after SNP calling (Figure 5A,C,D). These results were in agreement with the spiking of the samples.

#### 3.2.2. Metagenomics Analysis

The DNA extracts of the goat cheese samples were first tested with qPCR (results presented in Supplementary Materials Table S1). The presence of *E. coli* was detected in the DNA extracted from all spiked samples (*uidA*, Cq of 16 to 22). It was not detected in the blank. The virulence factors *eae* and *stx1*, harbored on the genomes of the two spiked strains, were detected with a Cq of 16 in the DNA extracted from all spiked goat cheese samples (results in Supplementary Materials Table S1).

**Figure 6:**
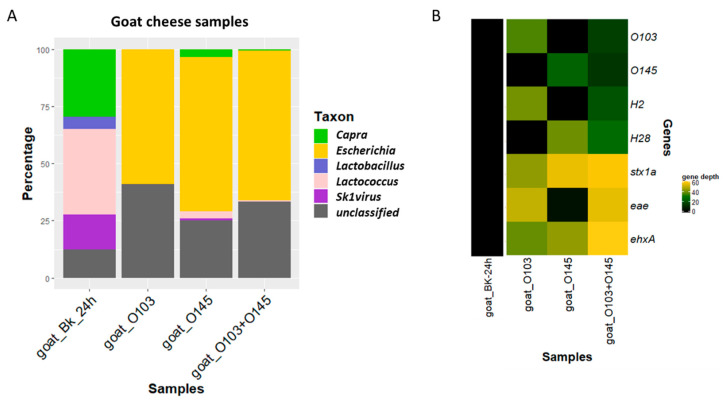
(**A**) Percentages of reads classified to the genus level using Kraken2 (taxonomic classification tool) on all reads of goat cheese samples with in-house databases of mammals, archaea, bacteria, fungi, human, protozoa, and viruses. Green represents the proportion of “*Capra*” corresponding to goat reads. Yellow represents the presence of “*Escherichia*” in the sample. The reads that could not be classified to the genus level for mammals, archaea, bacteria, fungi, human, protozoa, or viruses are represented in gray. (**B**) Gene depth per million trimmed reads per sample of *wzx* and *fliC* genes for the determination of types O103, O145, H2, and H28 and *stx1a, eae,* and *ehxA* virulence genes with more than 80% coverage and 80% identity in all reads of goat cheese samples. Increasing depth (per million trimmed reads) is represented in shades of green to yellow according to the color gradient in the legend. Goat_Bk_24h = Blank goat cheese enriched for 24 h. Goat_O103 = goat cheese spiked with STEC O103. Goat_O145 = goat cheese spiked with STEC O145. Goat_O103+O145 = goat cheese co- spiked with STEC O103 and STEC O145.

After sequencing, goat DNA (*Capra* genus) could be detected in the blank and in small percentages in DNA extracted from the goat cheese sample spiked with STEC O145 (3%) and co-spiked with STEC O103 and STEC O145 (0.6%), but not in the sample spiked only with STEC O103 (Figure 6A). More reads were unclassified in that sample. The blank was also composed of *Lactobacillus*, *Lactococcus,* and SK1virus (a *Lactoccocus* virus). Only some *Lactococcus* were still detected in the spiked samples, and more than 50% of the reads were classified as *Escherichia*, which is a genus that was not naturally present in the goat cheese before the spiking.

All genes of interest that were expected to be found linked to the spiked strain could be retrieved with a high depth in the sequencing reads of the DNA extracted from the spiked goat cheese samples (see Figure 6B). The subtype of the *stx1* gene could be identified without ambiguity and was identical for the two spiked strains, as expected from the analysis of the isolates using WGS. The depth or number of reads mapping to the virulence factors in the co-spiked sample was much higher than the number of reads mapping to serotyping genes in the co-spiked samples, which is as expected, because the virulence factors were to be found in the two strains, while both strains have a different serotype.

After strain-level metagenomics analysis, one strain was detected for each of the single spiked samples, while two were detected for the co-spiked sample. The strains were identified as STEC O103 and STEC O145 based on the detection of the serotyping alleles. These were placed in a phylogenetic tree in proximity of the genomes of sequenced isolates, including the spiked isolates (Figure 5A). Figure 5C depicts only the cluster of the STEC O103. The strains obtained from metagenomics samples and the corresponding spiked isolate were separated from the other sequenced isolates. The inferred STEC strain from the co-spiked sample had 2 SNPs difference per million genomic positions to the corresponding STEC O103 isolate, while the strain from the sample spiked only with STEC O103 had 1 SNP difference to this isolate (Supplementary Materials Table S3). The O-type, *stx1*, *eae, and ehxA* genes found in the isolate could be detected in the strains from the metagenomics samples. The spiked strain could be fully characterized using the reads obtained from the single as well as the co-spiked metagenomics samples. Interestingly, two isolates from the National Reference Laboratory (TIAC 1884 and TIAC 1878) were placed together in this cluster, with 1 SNP difference per million genomic positions (Supplementary Materials Table S2). These isolates come from food samples received at the same time period but that were not tested for relatedness at that time. Figure 5D details the cluster of the STEC O145. The inferred strains from metagenomics samples could be linked to the corresponding STEC O145 isolate with 2 and 0 SNPs difference per million genomic positions for the co-spiked and single spiked samples, respectively, and they could be separated from other sequenced isolates with the same range of SNPs distance as observed for TIAC 1220 to these other cases (Supplementary Materials Table S3). However, gene detection did not allow full characterization of the STEC O145, as *stx1* could not be detected in the inferred genome with the set parameters, although it was detected with a high depth in all reads from the samples (Figure 6B). However, *eae, ehxA,* and the serotype could be correctly detected, and the strain was placed in a cluster with the corresponding STEC strain, which indicates that there is a strong possibility that this strain is indeed a STEC. The reads containing the *stx1* gene were mapped to a separate sequence from the database when performing the Sigma workflow, which was not included in the reads corresponding to the STEC O145 strain.

## 4. Discussion

To rapidly confine foodborne outbreaks, it is important to be able to identify and characterize the food pathogen and to link it with the patient’s strain, in order to take appropriate measures to prevent further spreading as quickly as possible. Conventional microbiological detection methods are based on culturing steps to obtain an isolate of the pathogen. This increases the turnaround time of the analysis and is not always successful. Besides, these methods are based on low resolution technologies such as qPCR. Recently, with the advance of WGS, the resolution has been significantly increased, although an isolate is still needed. With shotgun metagenomics, this issue would be resolved, as all DNA of the sample is sequenced, and prior isolation is not needed. However, the difficulty lies then in the correct characterization of the pathogen and the subsequent source tracking based on the metagenomics reads, i.e., a mix of everything in the sample. There is a need to disentangle the reads of pathogens present in the food sample before being able to characterize these and to link these to the patient’s isolates. Moreover, the method should be adapted to an application in national reference and routine laboratories. Our study tested the performances of 5 sample preparation workflows for a short read shotgun metagenomics analysis of contaminated foods. The workflows were defined to be as close as possible to the standard methods currently used in many (reference) laboratories in Europe (isolation according to ISO/TS 13136:2012, followed by relatedness analysis in case of an outbreak). Therefore, we worked with very low loads of contamination (<10 CFU for 25 g of food) and enrichment media that fit the requirements of the ISO standard. Moreover, as other studies previously highlighted the need for an enrichment of the samples to obtain a high resolution in the analysis, we tested enrichment times approaching 24 h. Our results proved the feasibility of a metagenomics method to obtain the same information as the conventional methods in a manner that is relatively easily applicable in laboratories, and this in a shorter time period, as no isolation is needed. Time is a crucial factor during a foodborne outbreak investigation. This analysis was performed for samples containing multiple strains of *E. coli* and even several different strains of STEC. We also managed, for the first time, to link individual STEC strains from different food matrices containing multiple (including endogenous) *E. coli* strains to genomes from human cases, which is essential in resolving an outbreak.

In order to evaluate the possibility to implement our method as a new approach applicable in routine and demonstrate equal performance, we systematically compared the results obtained with metagenomics to the information collected using conventional methods. Therefore, our bioinformatics analysis was targeted at obtaining information comparable to that obtained with conventional methods. Such an approach has not yet been followed in other studies that were more focused on the research aspect for proof of concept. The possible presence of an STEC in the food sample was first evaluated with a taxonomic classification tool and the screening for virulence genes through all sequencing reads. This step corresponds to the screening stage in the conventional method (qPCR of specific virulence genes on the crude extract of the enriched test portion) and allows predicting if a potential pathogen is present in the sample. Although more sensitive tools exist [49], the taxonomic classification tool (Kraken2) was chosen for its fast execution (results in a few minutes) [33], which is appreciated for fast outbreak resolutions. Then, a strain-level classification of the metagenomics reads corresponding to the isolation of the strain was conducted. The obtained strain is characterized through gene detection and SNP phylogeny, which is equivalent to qPCR, followed by PFGE for relatedness or WGS analysis on an isolate in routine. However, the information obtained with the metagenomics analysis exceeded the one obtained with the conventional workflow. Indeed, information that is not requested in the scope of the ISO standard was also obtained for three different STEC serotypes tested: the metagenomics method was capable of distinguishing the subtype of the *stx1* and *stx2* genes but also to detect the gene *ehxA*, which are all recognized as markers for the severity of the disease but not included in the regulations [50]. Shotgun metagenomics, such as whole genome sequencing, allows obtaining an overview of the complete genome of the organisms present in a sample, therefore giving access to all genes of interest present on this genome. In further studies, other genes of interest including antimicrobial resistance genes but also other virulence genes recognized for their importance in other outbreaks such as *aaiC* or *aggR* (not present in the spiked strains) could also be investigated. Obtaining the complete genome of the STEC strain also allows the detection of any serotype, while only O26, O103, O111, O145, and O157 are currently looked for with the current methods.

The use of molecular methods in routine for food monitoring requires a DNA extraction protocol that is easy to implement with low costs and reproducible results. Therefore, we tested three commercial DNA extraction kits and two different food matrices, including one that is recognized as difficult due its higher fat content. All methods performed sufficiently well to detect and characterize the pathogenic strain in the food matrix and to link it to other outbreak cases from food and human origin. However, as previously shown, the choice of the kit can have a minor impact on the results obtained with a metagenomics study [11,12]. Indeed, it can cause a variation in the distributions and even the detection of genera in the sequencing reads of the same sample. However, the presence of some taxa could also be explained by carry-over or index misidentification due to the used sequencing technology [51] or performance of the taxonomy classification tool [33]. It has also been previously described that commercial DNA extraction kits can have different performances for the extraction of plasmid DNA [52], as observed in our study for the gene *ehxA*. As Nucleospin Food (workflow A) had good results for a low price and hands-on time and proved to be reproducible, it was selected for further experiments.

ISO/TS 13136:2012 demands an enrichment of the food matrix before the start of the analysis in order to be able to detect low levels of STEC. Moreover, implementation in a routine laboratory setting requires that the timing of the enrichment is practical for the technicians’ work schedule and allows a fast analysis, which is especially important in case of outbreak investigation. Previous studies have stressed the importance of an enrichment for metagenomics analysis of food samples and have focused on a shortening of the incubation time by using selective media or antibiotics [13,14]. In our study, the enrichment culture was conducted in the broths recommended in the ISO standard: the goat cheese was enriched in modified tryptic soy broth with acriflavin, and a non-selective broth (buffered peptone water) was used for the analysis of spiked beef, as it is recommended for stressed cells. The use of selective media has been shown to induce the identification of only certain serogroups of STEC [53]. We analyzed three different serotypes of STEC and noted no difference in the performances of our workflow. Two enrichment times were tested in our study (i.e., 24 h and 16 h). The information obtained after 16 h of enrichment, with the applied sequencing conditions, was already sufficient for outbreak investigation purposes with similar results to those obtained with the conventional methods, and we were able to conduct this analysis to the SNP level with the presence of endogenous *E. coli* in the food matrix. A DNA amplification, after 16 h of enrichment in non-selective broth, was not considered as an added value in the protocol, in contrast to what was previously reported after 12 h of incubation in selective broth followed by selective immune-based enrichment of the pathogen’s DNA [14]. Although a shorter enrichment time can be considered, we chose to pursue our study with an enrichment of 24 h, corresponding to what is currently performed in routine and is also recommended for the analysis of STEC under stress conditions [54].

In routine, the isolation of a strain almost automatically involves the characterization of a single pathogenic strain present in the food matrix for relatedness. However, previous studies have shown the prevalence of co-contaminations, including several different pathogens or multiple strains of the same species [55,56]. Therefore, it is important to develop a method that can characterize all pathogenic strains in the food vehicle for outbreak investigation. In our study, we managed to characterize STEC in food in the presence of endogenous non-pathogenic *E. coli* and in a sample co-contaminated with two different strains (serotypes O103 and O145), which is a level of analysis that was not achieved in previous studies [15]. We were able to extract the pathogen’s reads and link them back to human cases using phylogenetics analysis, starting from very low levels of inoculum. This information might not be obtained for two separate strains in a routine setting, as only one STEC (when the same profile of virulence genes of single colonies is obtained by qPCR) is usually characterized for relatedness after isolation. Moreover, as metagenomics is a “pathogen-agnostic” approach, it allows the analysis of a food product without the need of *a priori* knowledge on the pathogen or the number of strains that might be present. The conventional methods of analysis of foodborne outbreak samples rely on symptom-based screening for a pathogen. In one out of four foodborne outbreaks, the causative agent cannot be identified with the current method [57]. This can be caused by the absence of leftover suspect food, by a limited quantity of leftover food impeding the ability to conduct several conventional tests, or due to the difficulty to obtain an isolate to characterize further. The implementation of a metagenomics approach would allow a complete screening of possible pathogens in one test with a limited amount of sample. This might lead to interesting results as shown with the detection of other species in the enriched beef and goat, including *Hafnia*, which is a rare possible source of infection to humans, for example in immunocompromised patients. Although this study has focused on STEC as a bacterial foodborne pathogen, we believe that the same approach could be applied to others with only minor modifications, such as the database used for read classification and gene detection.

The possibility to implement new approaches in routine settings will also depend on the cost of the analysis per sample. Metagenomics studies still represent a high investment for laboratories. Yet, metagenomics provides access to much more information at once than conventional tests, which, if all conducted in parallel, would also become expensive, in addition to requiring a large portion of sample and therefore risking missing the causing agent if only a small amount is available (which is common for leftovers from a suspect meal). The cost is primarily linked to the low number of metagenomics samples sequenced in one run, although the price of sequencing has dropped significantly in the last few years. The amount of samples sequenced might vary depending on the desired depth, and therefore, the level of information obtained per sample could be improved by sequencing at a higher depth, but then also at a higher analysis cost. The necessary depth could also be evaluated from the qPCR result of specific markers of the pathogen in the food matrix, if it is known. However, although our metagenomics and qPCR results seemed to agree, other studies have shown that qPCR results are not directly linked to metagenomics outcome [58]. Moreover, in case of an outbreak, the time to wait for the accumulation of sufficient samples to start a run can be an obstacle for a fast response, although metagenomics runs require fewer samples than the WGS of isolates before the run is complete. To reduce the number of samples in a full run, new options with lower output such as the Flongle flow cell from Oxford Nanopore Technologies still have to be investigated and might prove cost-effective. The use of a long reads sequencing technology could offer additional advantages such as reducing the turn-around time by allowing real-time analysis and preventing bleed-through by sequencing one sample per flow cell. It might also improve the reconstruction of the genomes and the species detection by bringing bigger pieces to the puzzle [59]. However, the error rate of Oxford Nanopore Technologies is still relatively high and might impact the level of details obtained in the analysis, as observed by Hyeon et al. [14]. Another important drawback for the implementation of metagenomics in routine is the need for adapted bioinformatics pipelines [60]. This has been improving in the last years with the development of new specialized tools that can be proposed in workflows such as the one presented in this study. In the future, this workflow should be implemented as a user-friendly analysis pipeline to be executed in a routine setting. This will be worthwhile, as metagenomics approaches are now increasingly being explored, and studies such as this one prove their applicability for routine laboratories.

Interestingly, our analysis was also able to detect an unanticipated link between two isolates from the Belgian NRL received during the same time period. Relatedness and typing analyses represents extra tests and therefore extra costs, and these are not performed on a standard basis for all food isolates outside outbreak investigations in a reference or routine laboratory, while it could be achieved for every sample when performing metagenomics. This highlights the added value of the whole genome sequencing of pathogens in food samples, and by extension even from environmental samples. Importantly, this will also contribute to the creation and use of a shared database of whole genome sequences, including genomes of contaminants from human origin, in order to rapidly detect relationships between linked cases. This would allow to rapidly trace back the source of a contamination, similarly to what is being done using the genomic tracking tool GenomeTrakr [61]. The addition of pathogen whole genome sequences into a database could also improve our data analysis method, as Sigma, the strain-level inference tool used, is based on the use of reference genomes, for which 728 complete *E. coli* complete genomes were available in NCBI at the time of the analysis. The acquisition of circulating STEC genomes could help for the detection of strains less common in public databases such as the STEC O145 presented in this study, for which a virulence gene was missing after genome inference, although it could be detected with very high depth before the strain-level acquisition analysis. Although infrequently sequenced, STEC O145 is one of the top six most common non-O157 serotypes associated to human diseases [62], and it has previously been linked to a multi-strain outbreak in Belgium [63].

In conclusion, we presented a metagenomics method developed to be as close as possible to the actual ISO standard, but without requiring isolation. This study proved the applicability of metagenomics as a valid alternative to the standard protocols that are currently used in reference laboratories with a strain-level acquisition of reads replacing the isolation. We showed that this method can equal and even surpass the information that can be obtained with the conventional workflow, in one single test, allowing access to information on all genes in the DNA of the pathogen studied and the resolution of outbreaks by linking human cases to strains from food samples. However, the cost of the method, still high, might at first impose a rational use of the approach. The metagenomics method described in this study can be used as a faster alternative when urgent results are necessary, in particular in the case of outbreaks, or as an alternative to ISO for samples in which the isolate could not be obtained. It is also suitable to study emerging strains or pathogens such as the O104 strain from an international outbreak from German origin in 2011 [64] and would even provide the necessary sequence information to design a conventional method allowing the detection of the same strain from food for other laboratories that do not have the capacity to invest in a metagenomics approach. Moreover, the ability to discriminate and characterize several strains in case of multi-strain outbreaks is not yet covered in current procedures in routine but, as presented in this work, it can be achieved by following a metagenomics approach. New technologies allowing a metagenomics analysis at a lower cost and in an even shorter time-frame are yet to be explored further for a facilitated implementation in routine. This will only be feasible if guidelines are adapted to fit the methods that are being developed for public health and food chain safety needs. The possibility of applying whole genome sequencing and metagenomics for outbreak investigation, source attribution, and risk assessment of foodborne microorganisms has now been assessed by the EFSA [65], demonstrating the initiation of a reflection on future regulations in this matter. Studies such as ours can contribute to convincing the policy makers to adopt these new methods into practical procedures that may be applied in reference and routine laboratories in the near future.

## Supplementary Materials

The following are available online at https://www.mdpi.com/2076-2607/8/8/1191/s1, Table S1: Description of the samples and qPCR result on the DNA extract, Table S2: Gene detection in all metagenomics samples using SRST2, Table S3: SNP distances matrix (per million genomic positions) for STEC phylogenetic tree, Table S4: Model selection and parameters for all trees (Figure 5), Table S5: qPCR results on the enriched food samples and gene detection (SRST2) performed on the isolates obtained from the samples.
