# Supplementary material for: A Practical Method to Implement Strain-Level Metagenomics-Based Foodborne Outbreak Investigation and Source Tracking in Routine
Source: Microorganisms. 2020 Aug 5;8(8):1191. doi: 10.3390/microorganisms8081191 (PMC7463776; doi:10.3390/microorganisms8081191)
Supplement: Supplementary file 1 [file microorganisms-08-01191-s001.zip › sup_mat_1.pdf]

Sample ID	Description	Matrix	Biological replicate	Sequencing run *	CFU of STEC spiked in 25g food matrix	STEC spiked	Workflow	qPCR of the DNA extract			
								uidA	***eae***	***stx1***	***stx2***
N BK 0 -1	Blank beef not enriched, extracted with Nucleospin Food, biological replicate 1	Beef	1	1	0	-	-	33.27	ND	ND	ND
N Bk 24-1	Blank beef enriched 24h, extracted with Nucleospin Food, biological replicate 1	Beef	1	1	0	-	A	21.63	ND	ND	ND
N Bk 24-2	Blank beef enriched 24h, extracted with Nucleospin Food, biological replicate 2	Beef	2	2	0	-	A	18.33	ND	ND	ND
N Bk 24-3	Blank beef enriched 24h, extracted with Nucleospin Food, biological replicate 3	Beef	3	2	0	-	A	16.41	ND	ND	ND
beef_A-1	Beef spiked with 6 CFU of STEC enriched 24h, extracted with Nucleospin Food, biological replicate 1	Beef	1	1	6	TIAC 1152	A	20.46	21.56	21.19	22.01
beef_A-2	Beef spiked with 6 CFU of STEC enriched 24h, extracted with Nucleospin Food, biological replicate 2	Beef	2	2	6	TIAC 1152	A	19.34	19.27	19.3	20.51
beef_A1-3	Beef spiked with 6 CFU of STEC enriched 24h, extracted with Nucleospin Food, biological replicate 3, technical replicate 1	Beef	3	2	6	TIAC 1152	A	19.38	20.49	20.42	21.32
beef_A2-3	Beef spiked with 6 CFU of STEC enriched 24h, extracted with Nucleospin Food, biological replicate 3, technical replicate 2	Beef	3	2	6	TIAC 1152	A	19.39	20.14	20.08	21.23
beef_A3-3	Beef spiked with 6 CFU of STEC enriched 24h, extracted with Nucleospin Food, biological replicate 3, technical replicate 3	Beef	3	2	6	TIAC 1152	A	19.35	20.63	20.38	21.35
beef_B	Beef spiked with 6 CFU of STEC enriched 24h, extracted with Dneasy Blood&Tissue, biological replicate 1	Beef	1	1	6	TIAC 1152	B	18.67	19.82	19.42	20.69
beef_C	Beef spiked with 6 CFU of STEC enriched 24h, extracted with Zymo Research HostZero, biological replicate 1	Beef	1	1	6	TIAC 1152	C	17.78	18.72	18.64	20.06
beef_D	Beef spiked with 6 CFU of STEC enriched 16h, extracted with Nucleospin Food, biological replicate 3	Beef	3	2	6	TIAC 1152	D	19.53	20.38	20.20	21.5
beef_E	Beef spiked with 6 CFU of STEC enriched 16h, extracted with Nucleospin Food and amplified with phi29 DNA polymerase, biological replicate 3	Beef	3	2	6	TIAC 1152	E	22.8	23.97	24.76	25.33
goat_Bk_24	Blank goat cheese enriched 24h extracted with Nucleospin Food	Goat cheese	1	3	0	-	A	ND	ND	ND	ND
goat_O103	Goat cheese spiked with 7CFU STEC O103, enriched 24h and extracted with Nucleospin Food	Goat cheese	1	3	7	TIAC 1220	A	16.3	15.96	16.24	ND
goat_O145	Goat cheese spiked with 5CFU STEC O145, enriched 24h and extracted with Nucleospin Food	Goat cheese	1	3	5	TIAC 1878	A	22.25	15.9	15.94	ND
goat_O103+O145	Goat cheese spiked with 7CFU STEC O103 and 5 CFU STEC O145, enriched 24h and extracted with Nucleospin Food	Goat cheese	1	3	7-5	TIAC 1220 - TIAC 1878	A	16.74	15.46	15.7	ND

Table S1: Description of the samples and qPCR result on the DNA extract
ND: not detected after 40 qPCR cycles
*: Runs of 12 libraries, including other samples that are not presented in this study
