# Supplementary material for: A Practical Method to Implement Strain-Level Metagenomics-Based Foodborne Outbreak Investigation and Source Tracking in Routine
Source: Microorganisms. 2020 Aug 5;8(8):1191. doi: 10.3390/microorganisms8081191 (PMC7463776; doi:10.3390/microorganisms8081191)
Supplement: Supplementary file 1 [file microorganisms-08-01191-s001.zip › sup_mat_2.pdf]

Sample	Sequenced reads	Surviving reads after trimming	***wzx***				***flic***				***stx1***				***stx2***				***eae***			***ehxA***		
			Locus	% covered	% identity	depth	Locus	% covered	% identity	depth	Locus	% covered	% identity	depth	Locus	% covered	% identity	depth	% covered	% identity	depth	% covered	% identity	depth
**beef_Bk-0-1**	**2475845**	**2695013**	ND	ND	ND	ND	ND	ND	ND	ND	ND	ND	ND	ND	ND	ND	ND	ND	ND	ND	ND	ND	ND	ND
**beef_Bk-24-1**	**1637549**	**1637437**	ND	ND	ND	ND	ND	ND	ND	ND	ND	ND	ND	ND	ND	ND	ND	ND	ND	ND	ND	ND	ND	ND
**beef_Bk-24-2**	**1139280**	**1139214**	ND	ND	ND	ND	ND	ND	ND	ND	ND	ND	ND	ND	ND	ND	ND	ND	ND	ND	ND	ND	ND	ND
**beef_Bk-24-3**	**2695362**	**2695013**	ND	ND	ND	ND	ND	ND	ND	ND	ND	ND	ND	ND	ND	ND	ND	ND	ND	ND	ND	ND	ND	ND
**beef_A-1**	**1735479**	**1735304**	O157	88,11	100	5,1	H7	91,35	100	4,23	*stx1* a	76,85	100	4,11	*stx2* a	92,83	100	7,89	100	100	5,47	95	100	3,09
**beef_A-2**	**1153272**	**1153119**	O157	100	92	9,88	H7	100	100	8,24	*stx1* a	100	100	9,52	*stx2* a	100	100	15,86	100	100	13,51	100	100	9,18
**beef_A1-3**	**2317908**	**2317508**	O157	92,77	100	10,5	H7	97,78	99,77	7,57	*stx1* a	100	100	12,11	*stx2* a	100	100	13,24	100	100	12,79	96,9	100	6,3
**beef_A2-3**	**2937756**	**2937309**	O157	100	100	10,91	H7	100	100	11,24	*stx1* a	100	100	15,2	*stx2* a	100	100	16,81	100	100	21,1	99	100	10,03
**beef_A3-3**	**2204578**	**2204197**	O157	100	100	5,89	H7	98,46	99,48	9	*stx1* a	100	100	7,67	*stx2* a	100	100	10,24	100	100	11,85	98,06	100	4,99
**beef_B**	**1006641**	**1006503**	O157	89,8	100	1,96	H7	93	100	3,98	*stx1* a	97,31	100	2,31	*stx2* a	100	100	4,07	91,41	100	3,8	89,36	100	2,46
**beef_C**	**2269583**	**2269391**	O157	100	100	6,29	H7	100	100	13,26	*stx1* a	100	100	13,24	*stx2* a	100	100	11,37	100	100	15,57	100	100	29,67
**beef_D**	**2224794**	**2224472**	O157	100	100	5,44	H7	100	89	12,85	*stx1* a	100	100	9,45	*stx2* a	100	100	8,03	100	100	11,37	100	100	8,85
**beef_E**	**3307077**	**3306665**	O157	100	100	10,93	H7	100	89	15,52	*stx1* a	100	100	10	*stx2* a	100	100	11,84	100	100	14,39	100	100	8,37
**goat_Bk_24**	**2630580**	**2630000**	ND	ND	ND	ND	ND	ND	ND	ND	ND	ND	ND	ND	ND	ND	ND	ND	ND	ND	ND	ND	ND	ND
**goat_O103**	**2041482**	**2041209**	O103	100	100	72,39	H2	100	100	81,31	*stx1* a	100	100	87,98	ND	ND	ND	ND	100	100	102,1	100	100	78,52
**goat_O145**	**2715903**	**2715593**	O145	100	100	66,02	H28	83,97	98,15	106,6	*stx1* a	100	100	149,8	ND	ND	ND	ND	63	93,48	13,83	100	100	117,9
**goat_O103+O145**	**2919551**	**2919286**	O103	100	100	47,05	H2	100	100	61,64	*stx1* a	100	100	167,9	ND	ND	ND	ND	100	99,86	159,2	100	99,97	177,8
			O145	100	100	39,85	H28	83,97	98,15	80,18					ND	ND	ND	ND						

Table S2: Gene detection in all metagenomics samples using SRST2
ND: not detected
