# Supplementary material for: A Practical Method to Implement Strain-Level Metagenomics-Based Foodborne Outbreak Investigation and Source Tracking in Routine
Source: Microorganisms. 2020 Aug 5;8(8):1191. doi: 10.3390/microorganisms8081191 (PMC7463776; doi:10.3390/microorganisms8081191)
Supplement: Supplementary file 1 [file microorganisms-08-01191-s001.zip › sup_mat_3.pdf]

	O103-EH1717	O103-EH1783	O103-EH1831	O103-TIAC1878	O103-TIAC1884	O103-TIAC1885	O113-TIAC1660	O145-EH1533	O145-EH1846	O145-TIAC1220	O145-TIAC1596	O157-TIAC1151	O157-TIAC1152	O157-TIAC1153	O157-TIAC1165	O157-TIAC1169	O157-TIAC1638
O103-EH1717	0	28	128	97	85	111	2327	9443	9586	9634	9431	9522	8267	9625	6265	9387	7532
O103-EH1783	27	0	129	71	61	90	2353	9721	9862	9924	9692	9760	8456	9853	6380	9610	7729
O103-EH1831	129	133	0	120	104	105	2368	9716	9834	9877	9656	9797	8490	9888	6437	9632	7758
O103-TIAC1878	99	75	122	0	1	91	2383	9796	9881	9966	9727	9887	8589	9999	6470	9735	7832
O103-TIAC1884	93	69	113	1	0	85	2340	9501	9608	9669	9450	9324	8100	9407	6190	9179	7412
O103-TIAC1885	120	100	112	95	83	0	2333	9513	9612	9672	9451	9404	8178	9439	6198	9232	7501
O113-TIAC1660	4203	4372	4245	4195	3850	3922	0	10474	10565	10589	10399	10809	9521	10897	7322	10654	8825
O145-EH1533	8824	9343	9011	8921	8087	8275	5418	0	498	489	491	7759	6760	7844	5097	7622	6177
O145-EH1846	8874	9389	9035	8914	8101	8282	5414	493	0	60	17	7693	6682	7726	5033	7535	6089
O145-TIAC1220	8892	9420	9047	8964	8128	8309	5410	483	60	0	56	7671	6678	7743	4999	7507	6058
O145-TIAC1596	8824	9327	8966	8870	8054	8231	5386	491	17	56	0	7581	6610	7635	4970	7434	6011
O157-TIAC1151	7910	8339	8076	8004	7055	7272	4970	6897	6903	6904	6730	0	0	131	0	0	33
O157-TIAC1152	7890	8300	8041	7988	7041	7265	5030	6903	6889	6906	6742	0	0	131	0	0	33
O157-TIAC1153	8064	8490	8221	8164	7179	7361	5054	7032	6993	7029	6836	132	115	0	95	132	104
O157-TIAC1165	7727	8094	7879	7777	6954	7115	4999	6727	6706	6680	6552	0	0	140	0	0	34
O157-TIAC1169	7915	8333	8060	7999	7049	7245	4973	6877	6863	6858	6699	0	0	133	0	0	32
O157-TIAC1638	7745	8174	7917	7849	6942	7180	5023	6796	6764	6750	6605	41	35	127	28	39	0
beef_B_STECO157	7605	7985	7714	7656	6745	6989	4817	6607	6575	6617	6412	0	0	125	0	0	31
beef_C_STECO157	8042	8484	8200	8129	7150	7367	4999	7007	6991	7027	6843	0	0	132	0	0	32
beef_D_STECO157	8103	8530	8271	8203	7199	7442	5065	6959	6939	6961	6794	0	0	130	0	0	32
beef_E_STECO157	8065	8458	8226	8150	7179	7407	5043	6970	6935	6942	6759	0	0	140	0	0	32
beef_A-2_STECO157	7894	8296	8042	7986	6998	7236	4905	6917	6891	6899	6742	0	0	129	0	0	31
beef_A-1_STECO157	7658	8062	7775	7740	6806	7031	4873	6748	6729	6732	6556	1	0	137	0	1	34
beef_A1-3_STECO157	7853	8283	8003	7956	7000	7210	4882	6829	6843	6871	6680	0	0	125	0	0	31
beef_A2-3_STECO157	7864	8280	7996	7945	6986	7192	4910	6894	6890	6921	6728	0	0	128	0	0	31
beef_A3-3_STECO157	7880	8287	7996	7945	6983	7216	4895	6852	6856	6855	6666	0	0	127	0	0	27
beef_isolate_O157-1	7890	8340	8080	8025	7057	7267	4989	6913	6899	6923	6744	0	0	133	0	0	33
beef_isolate_O157-2	7911	8327	8061	7992	7074	7249	4996	6938	6909	6912	6738	0	0	136	0	0	31
beef_isolate_O157-3	7914	8320	8067	8001	7036	7255	4922	6894	6879	6909	6734	0	0	133	0	0	34
goat_O103+O145_STECO103	105	76	125	2	2	86	2466	10286	10402	10467	10224	10451	9062	10521	6831	10271	8246
goat_O103+O145_STECO145	8976	9511	9136	9067	8194	8387	5445	473	54	2	50	8075	7020	8142	5269	7911	6364
goat_O103_STECO103	108	73	130	1	1	96	2628	10333	10442	10484	10270	11099	9744	11162	7621	10949	9048
goat_O145_STECO145	8989	9533	9146	9082	8222	8406	5476	477	60	0	53	8144	7089	8209	5328	7996	6449
goat_isolate_O103	100	75	123	0	1	91	2383	9797	9882	9966	9728	9887	8588	9999	6470	9735	7833
goat_isolate_O145	8891	9419	9047	8964	8128	8309	5410	482	60	0	55	7671	6679	7743	5000	7507	6059
reference	7328	7730	7471	7416	6511	6747	4512	6392	6375	6393	6227	30	25	114	20	31	44

	beef_B_STECO157	beef_C_STECO157	beef_D_STECO157	beef_E_STECO157	beef_A-2_STECO157	beef_A-1_STECO157	beef_A1-3_STECO157	beef_A2-3_STECO157	beef_A3-3_STECO157	beef_isolate_O157-1	beef_isolate_O157-2	beef_isolate_O157-3	goat_O103+O145_STECO103	goat_O103+O145_STECO145	goat_O103_STECO103	goat_O145_STECO145	goat_isolate_O103	goat_isolate_O145	reference
O103-EH1717	4951	9303	8115	8675	8487	5299	8240	9431	8584	9214	8852	9642	104	10320	70	10242	97	9634	11099
O103-EH1783	5055	9543	8306	8846	8672	5424	8451	9655	8777	9469	9059	9857	73	10632	46	10561	71	9923	11383
O103-EH1831	5061	9558	8346	8916	8713	5421	8462	9663	8776	9507	9088	9904	125	10584	84	10499	120	9877	11401
O103-TIAC1878	5115	9650	8430	8996	8810	5496	8566	9778	8880	9616	9176	10003	2	10697	1	10617	0	9966	11525
O103-TIAC1884	4822	9082	7916	8478	8261	5171	8065	9200	8351	9047	8689	9412	2	10343	1	10285	1	9669	10826
O103-TIAC1885	4888	9156	8007	8559	8357	5227	8128	9267	8444	9117	8714	9497	91	10360	66	10289	95	9671	10979
O113-TIAC1660	5665	10448	9164	9798	9527	6092	9254	10638	9633	10525	10098	10833	4420	11309	3050	11271	4195	10589	12343
O145-EH1533	4020	7574	6513	7005	6950	4364	6696	7727	6975	7544	7254	7849	9536	508	6204	508	8921	488	9047
O145-EH1846	3963	7487	6433	6905	6858	4310	6647	7650	6913	7458	7156	7759	9553	57	6210	63	8914	60	8939
O145-TIAC1220	3976	7503	6434	6892	6846	4300	6654	7661	6892	7461	7138	7769	9583	3	6217	1	8963	0	8937
O145-TIAC1596	3906	7406	6366	6802	6782	4245	6559	7551	6795	7369	7054	7677	9491	54	6174	56	8870	56	8825
O157-TIAC1151	0	0	0	0	0	0	0	0	0	0	0	0	8613	7712	5924	7707	8004	6904	38
O157-TIAC1152	0	0	0	0	0	0	0	0	0	0	0	0	8579	7704	5975	7708	7988	6907	37
O157-TIAC1153	68	128	109	126	117	80	110	128	116	130	127	136	8745	7843	6009	7836	8163	7029	144
O157-TIAC1165	0	0	0	0	0	0	0	0	0	0	0	0	8359	7472	6039	7487	7776	6681	37
O157-TIAC1169	0	0	0	0	0	1	0	0	0	0	0	0	8591	7669	5931	7681	7998	6858	40
O157-TIAC1638	21	38	33	35	35	24	33	38	30	39	36	43	8412	7523	5978	7556	7849	6750	69
beef_B_STECO157	0	0	0	0	0	0	0	0	0	0	0	0	8251	7371	5701	7387	7655	6616	37
beef_C_STECO157	0	0	0	0	0	1	0	0	0	0	0	0	8753	7839	5963	7832	8129	7028	37
beef_D_STECO157	0	0	0	0	0	0	0	0	0	0	0	0	8815	7800	6004	7799	8202	6961	35
beef_E_STECO157	0	0	0	0	0	0	0	0	0	0	0	0	8750	7775	5995	7754	8150	6942	37
beef_A-2_STECO157	0	0	0	0	0	0	0	0	0	0	0	0	8579	7722	5818	7730	7985	6899	36
beef_A-1_STECO157	0	1	0	0	0	0	0	0	1	1	1	0	8318	7454	5797	7459	7740	6732	43
beef_A1-3_STECO157	0	0	0	0	0	0	0	0	0	0	0	0	8555	7655	5833	7657	7955	6870	37
beef_A2-3_STECO157	0	0	0	0	0	0	0	0	0	0	0	0	8536	7733	5801	7727	7945	6920	37
beef_A3-3_STECO157	0	0	0	0	0	1	0	0	0	0	0	0	8527	7648	5788	7648	7944	6855	34
beef_isolate_O157-1	0	0	0	0	0	1	0	0	0	0	0	0	8620	7722	5922	7733	8024	6923	40
beef_isolate_O157-2	0	0	0	0	0	0	0	0	0	0	0	0	8607	7729	5953	7729	7992	6912	39
beef_isolate_O157-3	0	0	0	0	0	0	0	0	0	0	0	0	8599	7704	5911	7703	8001	6910	42
goat_O103+O145_STECO103	5415	10207	8899	9486	9298	5802	9049	10320	9363	10146	9707	10561	0	11238	1	11177	2	10467	12169
goat_O103+O145_STECO145	4174	7887	6794	7273	7220	4486	6986	8066	7246	7843	7522	8164	9696	0	6263	0	9067	2	9425
goat_O103_STECO103	5777	10736	9358	10036	9736	6243	9526	10829	9813	10764	10367	11210	2	11208	0	11176	1	10484	12643
goat_O145_STECO145	4221	7952	6856	7320	7295	4530	7052	8134	7312	7926	7590	8238	9732	0	6303	0	9082	0	9498
goat_isolate_O103	5115	9650	8429	8996	8810	5496	8566	9778	8880	9615	9176	10003	2	10697	1	10618	0	9966	11524
goat_isolate_O145	3976	7503	6434	6892	6846	4299	6654	7660	6891	7461	7137	7770	9583	3	6217	1	8963	0	8937
reference	16	28	23	27	25	19	25	29	25	31	29	33	7971	7155	5363	7145	7414	6394	0

Table S3: SNP distances matrix (per million genomic position) for STEC phylogenetic tree
