# Supplementary material for: A Practical Method to Implement Strain-Level Metagenomics-Based Foodborne Outbreak Investigation and Source Tracking in Routine
Source: Microorganisms. 2020 Aug 5;8(8):1191. doi: 10.3390/microorganisms8081191 (PMC7463776; doi:10.3390/microorganisms8081191)
Supplement: Supplementary file 1 [file microorganisms-08-01191-s001.zip › sup_mat_4.pdf]

Tree	Model	#Param	BIC	AICc	lnL	Invariant	Gamma	R	Freq A	Freq T	Freq C	Freq G	A=>T	A=>C	A=>G	T=>A	T=>C	T=>G	C=>A	C=>T	C=>G	G=>A	G=>T	G=>C
SNP tree of all STEC strains	GTR	77,00	691312,49	690341,04	-345093,52	n/a	n/a	6,94	0,24	0,24	0,26	0,26	0,02	0,02	0,23	0,02	0,23	0,02	0,02	0,21	0,00	0,21	0,02	0,00
SNP tree of all STEC O157 strains	K2	34,00	5476,13	5229,85	-2580,81	n/a	n/a	4,24	0,25	0,25	0,25	0,25	0,02	0,02	0,20	0,02	0,20	0,02	0,02	0,20	0,02	0,20	0,02	0,02
SNP tree of all STEC O145 strains	K2	12,00	15956,54	15865,12	-7920,55	n/a	n/a	5,15	0,25	0,25	0,25	0,25	0,02	0,02	0,21	0,02	0,21	0,02	0,02	0,21	0,02	0,21	0,02	0,02
SNP tree of all STEC O103 strains	GTR	23,00	9966,48	9806,79	-4880,32	n/a	n/a	2,34	0,21	0,21	0,28	0,30	0,03	0,07	0,21	0,03	0,19	0,06	0,05	0,15	0,00	0,15	0,05	0,00

Table S4: Model selection and parameters for all trees (Figure 5)
GTR: General Time Reversible Model (with uniform rates)
K2: Kimura 2-parameters model (with uniform rates)
