# Supplementary material for: A Practical Method to Implement Strain-Level Metagenomics-Based Foodborne Outbreak Investigation and Source Tracking in Routine
Source: Microorganisms. 2020 Aug 5;8(8):1191. doi: 10.3390/microorganisms8081191 (PMC7463776; doi:10.3390/microorganisms8081191)
Supplement: Supplementary file 1 [file microorganisms-08-01191-s001.zip › sup_mat_5.pdf]

Sample	qPCR result crude extract from enriched food sample				gene detection isolate on WGS data				
	uidA	***eae***	***stx1***	***stx2***	***eae***	***stx1***	***stx2***	**O-type**	**H-type**
Beef_Bk-0h	ND	ND	ND	ND	N/A	N/A	N/A	N/A	N/A
Beef_Bk-24h-1	25,33	ND	ND	ND	N/A	N/A	N/A	N/A	N/A
Beef_Bk-24h-2	22,39	ND	ND	ND	N/A	N/A	N/A	N/A	N/A
Beef_Bk-24h-3	24,67	ND	ND	ND	N/A	N/A	N/A	N/A	N/A
Beef_24h -1	24,17	25,62	27,8	27,8	+	+	+	157	7
Beef_24h -2	22,42	22,07	23,95	23,64	+	+	+	157	7
Beef_24h -3	29,28	32,25	36,24	35,09	+	+	+	157	7
Beef_16h - 3	24,62	25,16	28,13	27,2	+	+	+	157	7
Goat_bk	ND	ND	ND	ND	N/A	N/A	N/A	N/A	N/A
Goat_O103	16,3	15,96	16,24	0	+	+	N/A	103	2
Goat_O145	22,25	15,9	15,94	0	+	+	N/A	145	28
Goat_O103+O145	16,74	15,46	15,7	0	+	+	N/A	103	2
					+	+	N/A	145	28

Table S5: qPCR results on the enriched food samples and gene detection (SRST2) performed on the isolates obtained from the samples.
N/A: not applicable (no isolate obtained or gene not present in the strain). +: detection of the gene with a minimum of 80% of coverage and depth of 5
ND: not detected after 40 qPCR cycles
